# Whole-Tissue Three-Dimensional Imaging of Rice at Single-Cell Resolution

**DOI:** 10.3390/ijms23010040

**Published:** 2021-12-21

**Authors:** Moeko Sato, Hiroko Akashi, Yuki Sakamoto, Sachihiro Matsunaga, Hiroyuki Tsuji

**Affiliations:** 1 Kihara Institute for Biological Research, Yokohama City University, Maioka 641-12, Totsuka, Yokohama 244-0813, Kanagawa, Japan; n175255d@yokohama-cu.ac.jp (M.S.); hakashi@yokohama-cu.ac.jp (H.A.); 2 Imaging Frontier Center, Organization for Research Advancement, Tokyo University of Science, 2641 Yamazaki, Noda 278-8510, Chiba, Japan; yuki_sakamoto@bio.sci.osaka-u.ac.jp (Y.S.); sachi@edu.k.u-tokyo.ac.jp (S.M.); 3 Department of Biological Sciences, Graduate School of Science, Osaka University, Machikaneyama-cho 1-1, Toyonaka 560-0043, Osaka, Japan; 4 Department of Applied Biological Science, Faculty of Science and Technology, Tokyo University of Science, 2641 Yamazaki, Noda 278-8510, Chiba, Japan; 5 Department of Integrated Biosciences, Graduate School of Frontier Sciences, The University of Tokyo, 5-1-5 Kashiwanoha, Kashiwa 277-8562, Chiba, Japan

**Keywords:** three-dimensional imaging, shoot apical meristem, root tip, rice

## Abstract

The three-dimensional (3D) arrangement of cells in tissues provides an anatomical basis for analyzing physiological and biochemical aspects of plant and animal cellular development and function. In this study, we established a protocol for tissue clearing and 3D imaging in rice. Our protocol is based on three improvements: clearing with iTOMEI (clearing solution suitable for plants), developing microscopic conditions in which the Z step is optimized for 3D reconstruction, and optimizing cell-wall staining. Our protocol successfully 3D imaged rice shoot apical meristems, florets, and root apical meristems at cellular resolution throughout whole tissues. Using fluorescent reporters of auxin signaling in rice root tips, we also revealed the 3D distribution of auxin signaling events that are activated in the columella, quiescent center, and multiple rows of cells in the stele of the root apical meristem. Examination of cells with higher levels of auxin signaling revealed that only the central row of cells was connected to the quiescent center. Our method provides opportunities to observe the 3D arrangement of cells in rice tissues.

## 1. Introduction

The three-dimensional (3D) arrangement of cells in tissues provides an anatomical reference for monitoring the development and function of organisms [1]. For example, in the shoot apical meristem (SAM), the plant tissue that generates the above-ground organs, stem cells are located at the tip and differentiated cells are arranged around the peripheral zone that surrounds the stem cells [2]. In the root apical meristem (RAM), located at the root tip, stem cells are arranged around the quiescent center (QC) and differentiate into diverse cell types including the columella, stele, and endodermis [3]. The 3D arrangement of cells is important for understanding how the developmental processes of multicellular organisms function. 

Topology and anisotropy are crucial characteristics to understand the development of organs with complex cellular patterns [4]. The relationship between the topology of cells and anisotropy of tissues in plants has been investigated [5]. Mechanical stress in the cell wall is caused by the 3D arrangement of cells, which is essential for proper organ differentiation [6]. These studies provided important insights about the topology and anisotropy of developing organs in plants; however, these studies have been limited to the analysis of two-dimensional sections. Since plant tissues are organized in three dimensions, a three-dimensional analysis is necessary to understand topology and anisotropy precisely.

Recent advances in tissue clearing methods have provided opportunities to observe the 3D arrangement of cells in tissues. For animal tissues, various clearing methods, such as ScaleS [7], CUBIC [8], and CLARITY [9], have enabled whole-tissue or whole-body imaging at the cellular resolution. For plant tissues, ClearSee [10], TOMEI [11], and PEA-CLARITY [12] have been proposed as protocols for tissue clearing and 3D imaging. These protocols have been used successfully for imaging pollen–pistil interaction [10], embryogenesis [13], and vascular development [14]. Recently, ClearSeeAlpha [15] and iTOMEI (see methods) were reported as improved methods for observing fluorescent proteins in fixed plant tissues and expanded the variety of plant species that can be cleared.

Rice is an important staple crop that provides 50% of the calories that the entire human population consumes and is a staple crop in more than 100 countries. Productivity improvements for rice are likely to come from advances in rice genetics, biotic and abiotic stress responses, and developmental biology [16,17]. In addition, rice is a model plant for monocotyledons with a growing number of rice genomic sequences, wider genetic diversity, and a long history of detailed study [18,19,20]. To improve our understanding of rice developmental biology, observation of cellular arrangements in 3D is a powerful tool; however, studies using tissue clearing and 3D imaging of rice have not been widely used due to the lack of established protocols. Three-dimensional imaging at single-cell resolution would allow quantitative analysis of cellular differentiation with modeling programs, e.g., MorphographX [21].

In this study, we optimized a protocol for tissue clearing and 3D imaging in rice. Our protocol is based on three improvements: clearing with iTOMEI, developing microscopic conditions in which the Z step is optimized for 3D reconstruction, and optimizing cell-wall staining. We observed SAMs, florets, and RAMs at cellular resolution throughout whole tissues. Using this protocol, we also revealed the 3D distribution of fluorescent reporters of auxin signaling in the root tip of rice.

## 2. Results

### 2.1. Development of a Tissue Clearing and 3D Imaging Protocol in Rice

**Figure 1:**
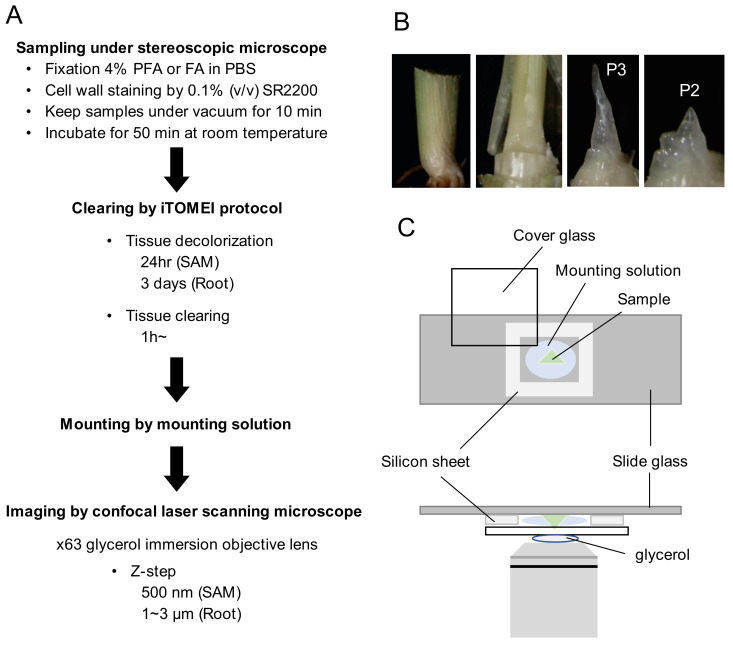
(**A**) Overview of the imaging protocol. (**B**) Sampling of a shoot apex. Far left: basal region of a seedling; middle left, after removing the outer three leaves; middle right, shoot apex with a P3 primordium; far right: shoot apex with a P2 primordium. (**C**) Mounting and preparation for imaging by confocal laser scanning microscopy. Upper: Samples were mounted on a silicon sheet with a square piece removed to maintain the 3D structure of tissues. Lower: a 63× glycerol-immersion objective lens was used. Bars; 100 μm.

To establish a protocol for tissue clearing and 3D imaging in rice, we improved methods for observation in four steps (Figure 1A). First, we optimized methods for sampling, fixation, and cell-wall staining of the dissected tissue. To sample SAMs, we carefully removed several leaves from the basal part of the seedlings by hand sectioning (Figure 1B). Then, the outermost P4 and P3 leaf primordia were removed with a scalpel while observing the samples with a stereoscopic microscope. The shoot apex, including leaf primordia P2 and P1 (plastochron number 2 and 1, which indicate the second youngest and youngest [20]) and the SAM, was exposed at this step and was excised by cutting at the middle of the stem. Excised shoot apices were placed into microtubes containing the fixative. This procedure enabled us to observe two leaf primordia and the SAM simultaneously. Occasionally, we sampled the shoot apex attached to the P3 leaf primordium. If necessary, P2 and P1 primordia were removed and only the SAM was sampled (Supplementary Materials Video S1). For fixatives, either 4% (*w*/*v*) paraformaldehyde or formaldehyde in phosphate-buffered saline can be used. For cell-wall staining, we used 0.1% (*v*/*v*) SCRI Renaissance 2200 (SR2200). 

Second, sampled tissues were cleared by iTOMEI (Figure 1A). Samples were placed in the decolorization solution of iTOMEI, followed by transfer to iTOMEI clearing solution. The time required for decolorization depended on the sample: 24 h for SAMs, 3 days for roots. 

Third, the samples were mounted. The selection of a mounting solution depends on the refractive index of immersion for the lens to be used for observation. We used iTOMEI clearing solution as the mounting solution because it was optimal for our observation conditions. To maintain the 3D structure of tissues, samples were mounted on a 0.2 mm thickness silicon sheet with a square (8 mm × 8 mm) removed from the center (Figure 1C). 

Fourth, the mounted samples were observed using a confocal laser scanning microscope equipped with a 63× glycerol-immersion objective lens. In order to collect a sufficient number and density of Z-stacked images for 3D reconstruction, we set the Z step at 500 nm for SAMs and 1–3 µm for root tips (Figure 1A).

### 2.2. Three-Dimensional Imaging of Rice SAMs with Single-Cell Resolution 

**Figure 2:**
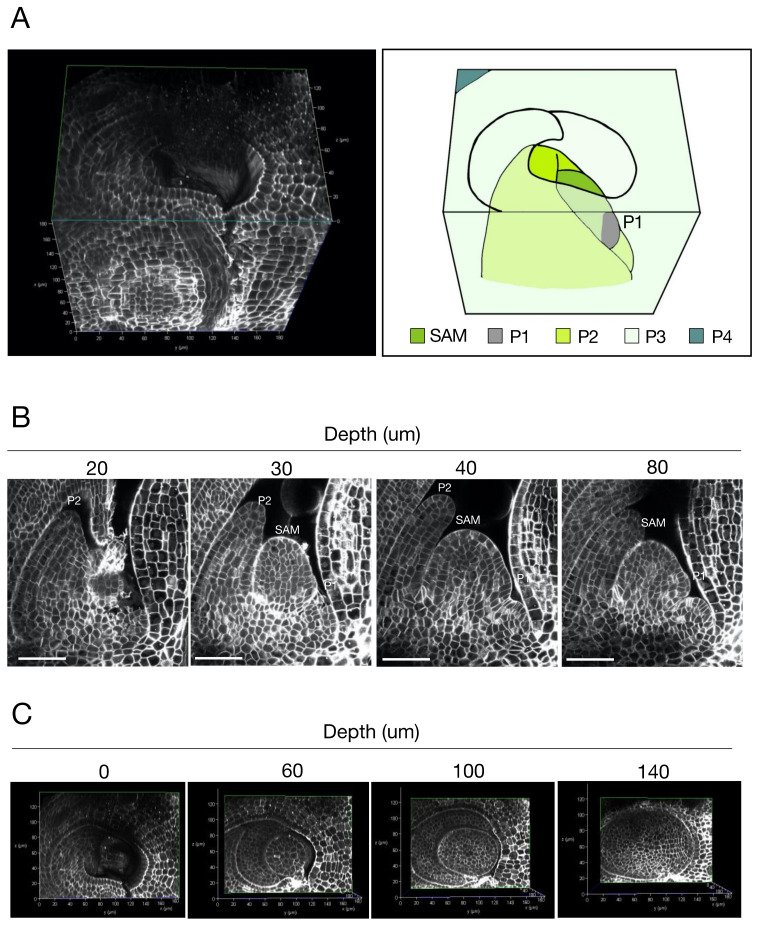
(**A**) Left: a 3D-reconstructed rice shoot apex. Right: a schematic presentation of the 3D-reconstructed shoot apex shown in the left panel. SAM; shoot apical meristem, P1; youngest leaf primordium (plastochron number 1), P2; second youngest leaf primordium (plastochron number 2), P3; third youngest leaf primordium (plastochron number 3), P4; fourth youngest leaf primordium (plastochron number 4). (**B**) Longitudinal sections of a rice SAM. Confocal optical sections were arranged according to the depth from the first section of serial imaging. The fluorescence intensity of cell-wall staining was adjusted for clear recognition of individual cells in the deeper sections. (**C**) Transverse sections of the 3D-reconstructed shoot apex. The transverse sections were arranged according to the depth from the top of the reconstructed image shown in (**A**). Bars; 50 μm.

We used our protocol to observe rice SAMs (Figure 2 and Supplementary Materials Video S2). We acquired longitudinal sections of SAMs at 500 nm Z steps; successfully obtained images of the entire tissue sample including the SAM, P1, P2, and part of P3; and reconstructed the 3D structure of the shoot apex (Figure 2A). We observed at cellular resolution that the SAM was wrapped around leaf primordia in a three-dimensional arrangement.

The SAM in rice is composed of the tunica and the corpus, two cell populations that are derived from different cell lineages [2]. The tunica is the outermost single cell layer and is called the L1. The corpus is an inner cell population inside the L1. To examine whether this cellular arrangement can be observed using our protocol, we acquired longitudinal sections through the whole tissue (Figure 2B, Supplementary Materials Figure S1 and Video S2). Increasing depth resulted in a decreased fluorescence intensity in cell-wall staining (Supplementary Materials Figure S1). We adjusted the fluorescence intensity of cell-wall staining in all images in Figure 2B for well-defined recognition of individual cells in the deeper sections and clearly observed the cellular arrangement of the SAM. The single-cell layer of the L1 was distinguished from the inner cells throughout the SAM. We also found that the second layer of cells from the outermost subepidermal layer tended to be aligned. 

We reconstructed the SAM in 3D from serial longitudinal sections and, using this reconstruction, we were able to obtain transverse sections of the same SAM (Figure 2C). We detected cell-wall staining throughout the transverse sections, enabling us to observe structures at cellular resolution. In summary, our protocol resulted in a series of images showing the precise cellular arrangement of the SAM and leaf primordia in 3D.

### 2.3. Three-Dimensional Imaging of Rice Florets with Single-Cell Resolution 

**Figure 3:**
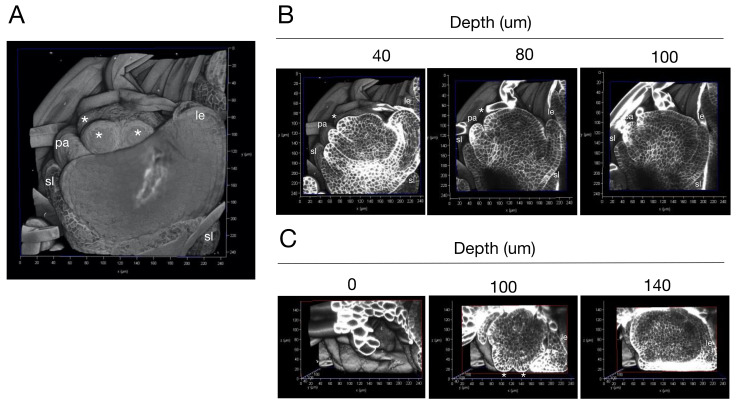
(**A**) A 3D-reconstructed rice floret. (**B**) Longitudinal sections of the floret. Confocal optical sections from the reconstructed image were arranged according to their depth from the first section of serial imaging. (**C**) Transverse sections of the 3D-reconstructed floret. The transverse sections were arranged according to the depth from the top of the reconstructed image shown in (**A**). sl; sterile lemma primordium, le; lemma primordium, pa; palea primordium, lo; lodicule primordium, *; stamen primordium.

Next, we used our protocol to observe rice florets (Figure 3 and Supplementary Materials Video S3). Rice florets are composed of the outermost structure, the lemma, and moving toward the interior, the palea, two lodicules, six stamens, and the innermost structure, the pistil, all generated by the floret meristem [20]. We observed the floret at the Sp5 stage when stamen primordia are developing. We acquired longitudinal sections of the floret and reconstructed a 3D image of the object (Figure 3A). Developing lemma and palea and four stamen primordia were visible from the front view, whereas the posterior side was covered by bract hair. To examine whether this cellular arrangement can be observed throughout the floret, we acquired longitudinal sections (Figure 3B and Supplementary Materials Video S3). Each cell was clearly distinguishable in the sections, and the internal cellular arrangement of the primordia for the lemma, palea, lodicule, and stamens was revealed. The same floret was observed from reconstructed images acquired from transverse sections (Figure 3C). We were able to observe the floret with cellular resolution from the apex to the base. The 3D arrangement of cells in the primordium for each organ was also found (Supplementary Materials Video S3).

### 2.4. Three-Dimensional Imaging of the Rice Anther with Single-Cell Resolution 

**Figure 4:**
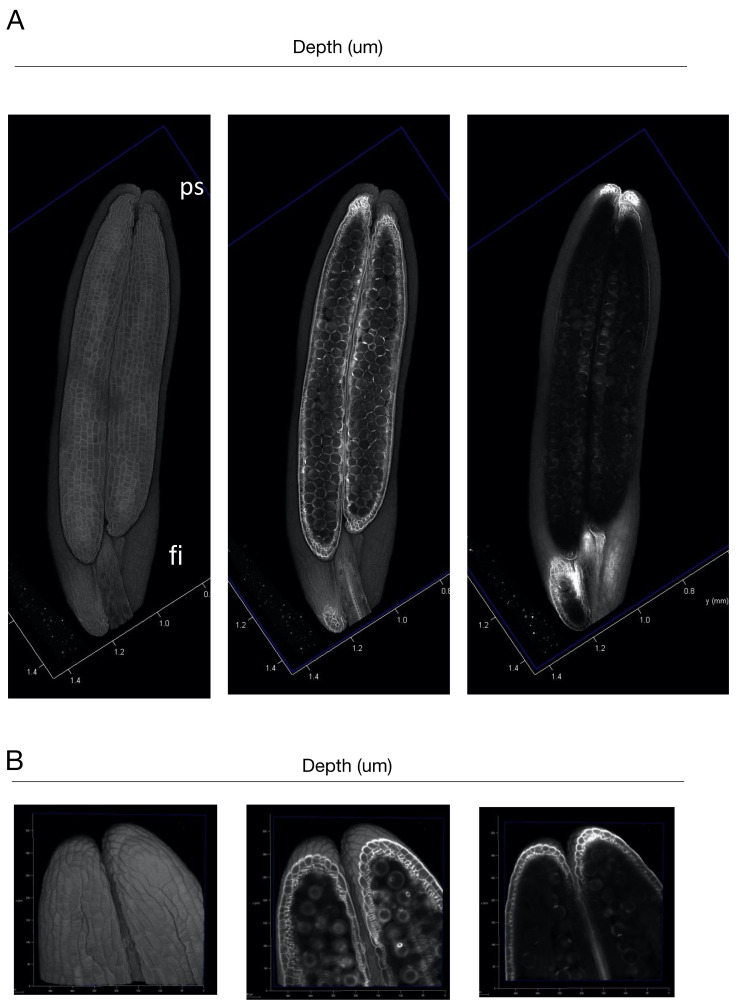
(**A**) Longitudinal sections of the 3D-reconstructed rice anther. (**B**) Longitudinal sections of the 3D-reconstructed rice tip of pollen sacs. ps; pollen sac, fl; filament, pg; pollen grain, gp; germination pore. Bars; 100 μm.

We examined how large organs can be observed based on this protocol. We used a mature anther, which is composed of four pollen sacs [22]. The mature pollen sac is approximately 1 mm long and 0.2 mm wide. Because the object to be scanned is large, it is necessary to use a ×20 lens. Thus, the anther was cleared with ClearSee, which has a refractive index suitable for this lens [10]. In order to create a single image of the entire object, the images were concatenated and reconstructed (Figure 4A and Supplementary Materials Video S4). As a result, the entire image of the pollen sacs could be observed. In the central section of the pollen sacs, we observed aligned epidermal cells, degenerated second layer, and germination pore of the pollen grains. When the tip of the pollen sacs was magnified with a ×40 lens, the structure of the pollen sacs and pollen grain could be clearly observed (Figure 4B and Supplementary Materials Video S5). In particular, the morphology of the second layer of cells in the pollen sacs could be observed more clearly.

### 2.5. Three-Dimensional Imaging of the Rice RAM with Single-Cell Resolution 

**Figure 5:**
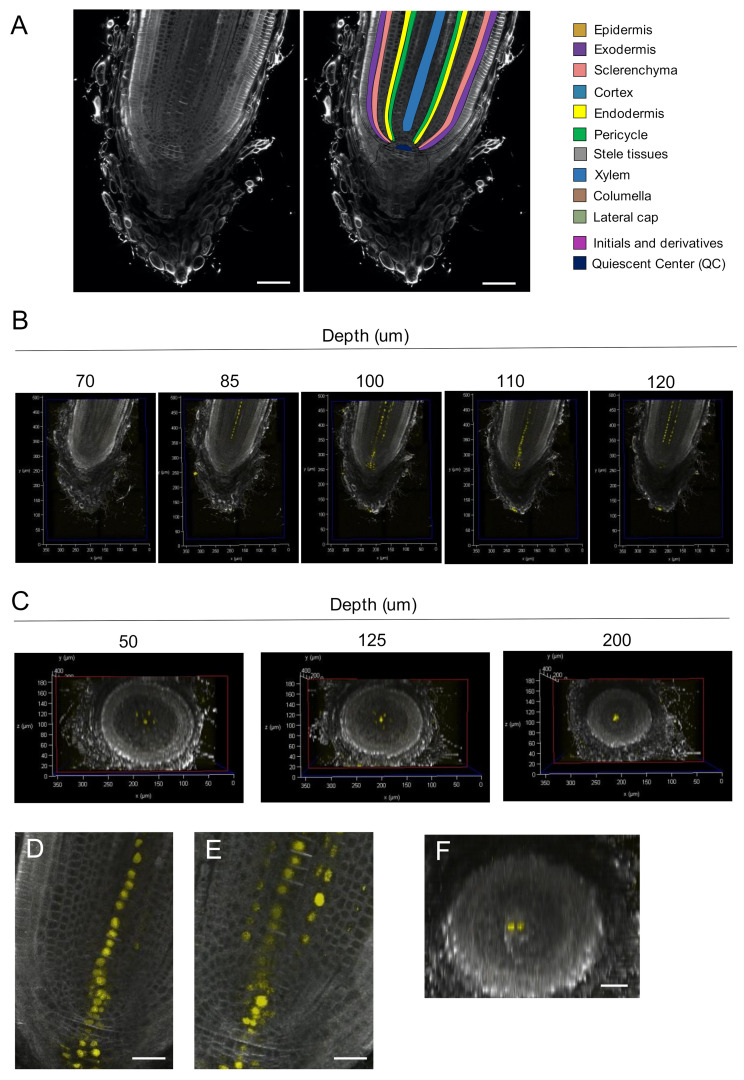
(**A**) Left: representative longitudinal section of a 3D-reconstructed rice root tip. Right: schematic presentation of the 3D-reconstructed root tip shown in the left panel. (**B**) Longitudinal sections of the root tip. Confocal optical sections from the reconstructed image were arranged according to the depth from the first section of serial imaging. (**C**) Transverse sections of the 3D-reconstructed root tip. The transverse sections were arranged according to the depth from the top of the reconstructed image. (**D**,**E**) Enlarged view of longitudinal sections with a central row of Venus-expressing cells connected to the QC (**D**) and with lateral rows of Venus-expressing cells that are not connected to the QC (**E**). (**F**) A transverse section across the QC. Bars; 50 μm in (**A**) and 25 μm in (**D**–**F**).

Next, we examined whether our method could be used to image roots and whether the method was suitable for fluorescence observations. Rice roots consist of several tissues; from the interior to the exterior, the tissues include the central stele with vascular bundles, the endodermis, the cortex, the sclerenchyma, the exodermis, and the epidermis [3]. The columella and the lateral root cap are located in the root tip. We acquired longitudinal sections of the root tip and confirmed that these tissues were distinguishable in our observations (Figure 5A,B). We reconstructed a 3D image of a root tip from serial longitudinal sections and examined the transverse sections (Figure 5C, Supplementary Materials Video S6). The transverse sections showed that organ shape was maintained in this orientation, and the positional relationship of the central-lateral axis was preserved; however, we could not distinguish each cell in the reconstructed transverse sections of root tips because the Z-step spacing was not sufficiently dense.

To examine whether we could observe the 3D distribution of fluorescent proteins, we used the auxin response reporter DR5rev:NLS-3xVenus [23,24]. Without clearing and cell-wall staining, the DR5 signal was observed in the stele and the columella cells, although precise observation at cellular resolution could not be achieved (Supplementary Materials Video 6).

We acquired longitudinal sections of rice root tips in which fluorescent cell-wall staining and Venus expression simultaneously occurred in a DR5rev:NLS-3xVenus plant (Figure 5B, Supplementary Materials Video S6). Venus fluorescence was observed in columella cells, the QC, and several rows of cells in the stele. In the columella and the QC, three and two interconnected cells expressed Venus, respectively. In the stele, there was a row of Venus-expressing cells in the center and lateral rows of Venus-expressing cells one to two cells away from the center. There were five lateral rows of cells (Figure 5C) corresponding to the positions of metaxylem in the differentiation zone. 

To determine whether the central and lateral rows of Venus-expressing cells were linked to the QC, we analyzed enlarged images of the sections (Figure 5D,E). The central row of Venus-expressing cells was linked to the QC. In contrast, the lateral rows of cells were not. Close inspection of the QC region revealed that there were two Venus-expressing cells at the QC (Figure 5F). These results suggest different origins of the central and lateral metaxylem.

## 3. Discussion

### 3.1. Tissue Clearing and 3D Imaging Enabled the Precise Evaluation of the Cellular Arrangement of Rice Tissues 

Resulting from this study, we propose a protocol to acquire images at cellular resolution of all layers of rice tissues. Using this protocol, we were able to obtain 3D reconstructed images. The advantages of this protocol can be summarized in five points. 

First, we were able to visualize the 3D reconstruction of tissues and the internal arrangement of cells across all layers simultaneously. Conventionally, we observed 3D images of tissues by SEM but not the internal cell arrangement. Conventional cross-sectioning is useful for observing the interior, but it cannot be used to construct a 3D structure. For example, it is difficult to appreciate the complexity of the 3D arrangement and shape of lodicule primordia because the tissues are surrounded by the lemma primordium and stamen primordia (Figure 3A). By examining successive sections while referring to the 3D constructed image in this study, it became easier to appreciate the arrangement and morphology of the lodicule in three dimensions (Figure 3B,C and Supplementary Materials Video S3).

Second, our protocol allows longitudinal and transverse sections to be observed simultaneously from the same tissue. In conventional sectioning, transverse sections could not be obtained after longitudinal sections were created. In the method optimized by this study, transverse sections were obtained from the same tissue by serially acquiring longitudinal sections and 3D reconstructions. This advantage gave us a three-dimensional view of the number and arrangement of the central and lateral rows of DR5-expressing cells at the root tip (Figure 5, Supplementary Materials Video S6 and discussed below).

Third, 3D construction allows for a continuous view of the internal structure. This contiguity evoked the possibility of specialization for the second cell layer of the SAM. Our data suggest that the second layer tends to be aligned (Figure 2). The composition of cells in grass SAMs has been dichotomized into the outermost layer of tunica cells and the inner layer of corpus cells with no special identity proposed for the second layer from the outside [2]. Our data indicate that the second layer is well aligned and distinguishable, suggesting that it may be specialized, although its function is unknown at present. 

Fourth, tissues with complex three-dimensional arrangements can be observed. It is difficult to observe accurate 3D arrangements of tissues that are wrapped together when using conventional sectioning methods. The method reported here enables us to acquire transverse sections continuously in the *Z*-axis direction, making it possible to observe the precise 3D arrangement of tissues. This advance made it possible to observe the arrangement of tissues, such as the leaf primordium in the SAM (Figure 2C, Supplementary Materials Video S2), the lemma, and the palea primordium (Figure 3C, Supplementary Materials Video S3).

Fifth, our 3D imaging method provides a unique opportunity to understand the mechanism for the maintenance of undifferentiated cells by supporting quantification of the velocity of cell replacement in the SAM and RAM. In the SAM, the organizing center is located at a certain distance from the stem cells. In the RAM, undifferentiated cells are maintained within a certain distance from the QC. Maintaining the proper distance between regions is essential to ensure the integrity of the SAM and RAM functions. Since the proper distance between regions is determined by the velocity of cell replacement, it is necessary to measure the velocity. So far, the velocity of cellular replacement has been measured on two-dimensional sections of the root tip of Arabidopsis [25]. The 3D imaging technique developed in this study will enable us to measure velocity comprehensively in three dimensions.

### 3.2. Spatial Distribution of DR5 Signals in the Root Tip 

The fate of cells that differentiate into metaxylem is determined by the activation of auxin signaling at the root tip [26]. The location of these cells at the root tip was not well defined in rice. Our results showed that DR5 signals are predicted to be in five locations of the RAM stele before metaxylem differentiation (Figure 5 and Supplementary Materials Video S6). 

Since diverse root cell types differentiate from stem cells in the vicinity of the QCs, rows of cells in which auxin signaling is activated and metaxylem differentiation occurs were thought to be connected to QCs. We found that in the root tip of a DR5rev:NLS-3xVenus plant, the central row of Venus-expressing cells was connected to the QC, but the other four rows of cells were not. The origin of the four unconnected DR5-expressing cells is not clear. DR5 expression may be repressed in the vicinity of the QC because DR5 fluorescence can be seen several cells away from the QC. Suppression of auxin signaling around the QC may be due to cytokinin localization because cytokinin is known to be distributed around the QC and is also known to inhibit auxin signaling [26,27,28].

## 4. Materials and Methods

### 4.1. Plant Materials and Growth Conditions

The Japonica rice cultivar Nipponbare was used for SAM and floret observations. The Japonica rice cultivar Norin 8 was used to generate DR5rev:NLS-3xVenus transgenic rice plants [23] and for observations of root tips. Plants were grown in climate-controlled chambers at 70% humidity under short-day conditions with daily cycles of 10 h of light at 28 °C and 14 h of dark at 25 °C. Light was provided by fluorescent white light tubes (400–700 nm, 100 μmol m^−2^·s^−1^). 

### 4.2. Sampling of Rice Shoot Apical Meristems and Florets

Plants of 25–35 days after germination were used to sample the SAMs. Slightly older plants of 35–45 days after germination were used to sample the florets. To sample SAMs, we carefully removed several leaves from the basal part of the seedlings by hand sectioning. The leaf primordia were removed carefully to expose the P2 leaf primordium as viewed with a stereoscopic microscope. The shoot apex was excised by cutting at a point 2–3 mm below the shoot apex, and the excised tissue was fixed in a microtube (Figure 1B, Supplementary Materials Video S1). 

### 4.3. Fixation and Cell-Wall Staining

The samples were fixed in 4% (*v*/*v*) paraformaldehyde or formaldehyde in PBS supplemented with 0.1% (*v*/*v*) SR2200 (the solution from the supplier was considered to be 100%: Renaissance Chemicals, UK). Samples in the fixative were vacuumed infiltrated for 10 min on ice. After restoration to normal pressure, the samples were incubated for 50 min at room temperature.

### 4.4. Clearing by the iTOMEI Protocol

The samples were transferred to a decolorization solution (100 mM sodium phosphate buffer at pH 8.0 with 20% (*w*/*v*) caprylyl sulfobetaine (TCI, Tokyo, Japan)) and incubated for 24 h for SAMs and florets or 3 days for roots at room temperature to decolorize the tissues [29]. The samples were transferred to a clearing solution (56.2% (*w*/*w*) Histodenz (Merck, Darmstadt, Germany) in PBS buffer) and incubated for 1 h at room temperature for SAMs, florets, and roots [29]. For anther, fixed anther was transferred to ClearSee and incubated for 1 week at room temperature. After incubation, the anther was mounted and observed.

### 4.5. Mounting Samples and Imaging by a Confocal Laser Scanning Microscope

The samples were mounted on glass slides using the same product we used as a clearing solution. Small samples were supported by a silicon sheet with holes (8 mm × 8 mm square). The anther was mounted with water. The samples were visualized with a confocal laser-scanning microscope (TCS SP8; Leica Microsystems, Tokyo, Japan) equipped with a 405 nm and pulsed white-light laser (WLL) sources and a 63× glycerol-immersion objective lens (HC PL APO 63×/1.30 GLYC CORR CS2; Leica Microsystems), a 40× water-immersion objective lens (PL APO CS2 40×/1.10 W CORR HCX; Leica Microsystems), and a 20× objective lens (PL APO CS2 20×/0.75 IMM CORR HC; Leica Microsystems). For SR2200 fluorescence, images were captured at 410–480 nm after excitation at 405 nm with a solid-state laser. For Venus fluorescence, images were captured at 520–600 nm after excitation at 515 nm with WLL. We set the Z step at 500 nm for SAMs and 1–3 µm for root tips. After image acquisition, which took approximately 2 h for 150 μm in depth, the images were processed using LASX software (Leica Microsystems, Tokyo, Japan).

## Data Availability

All datasets supporting the conclusions of this article are included in the article and supplementary files.

## Supplementary Materials

The following are available online at https://www.mdpi.com/article/10.3390/ijms23010040/s1.
