# Supplementary figures and images for: Whole-Tissue Three-Dimensional Imaging of Rice at Single-Cell Resolution
Source: Int J Mol Sci. 2021 Dec 21;23(1):40. doi: 10.3390/ijms23010040 (PMC8744978; doi:10.3390/ijms23010040)

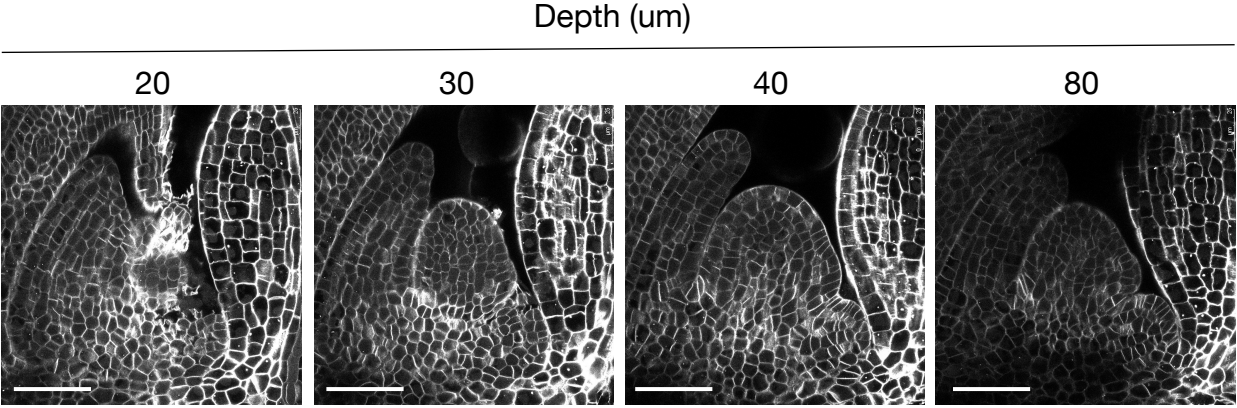

Supplementary Figure S1

Supplement: Supplementary file 1 [file ijms-23-00040-s001.zip › Figure_S1.pdf]
